# Circular RNA profiling identified an abundant circular RNA circTMTC1 that inhibits chicken skeletal muscle satellite cell differentiation by sponging miR-128-3p

**DOI:** 10.7150/ijbs.36412

**Published:** 2019-08-19

**Authors:** Xiaoxu Shen, Zihao Liu, Xinao Cao, Haorong He, Shunshun Han, Yuqi Chen, Can Cui, Jing Zhao, Diyan Li, Yan Wang, Qing Zhu, Huadong Yin

**Affiliations:** Farm Animal Genetic Resources Exploration and Innovation Key Laboratory of Sichuan Province, Sichuan Agricultural University, Chengdu, Sichuan 611130, PR China

**Keywords:** Skeletal muscle satellite cell, differentiation, circTMTC1, miR-128-3p, miRNA sponge, chicken

## Abstract

**Scope:** Myogenesis involves a series of complex cellular and developmental processes regulated by many genes, transcription factors and non-coding RNAs. Recent studies have demonstrated the involvement of circular RNAs (circRNAs) in myogenesis. While previous studies have established a role for some circRNAs, the precise functions and mechanisms of circRNAs in skeletal muscle development are still not completely understood in chicken.

**Methods:** To identify potential circRNAs during chicken embryonic skeletal muscle development, rRNA^-^ libraries sequencing was performed in breast muscles from 12 broilers and 12 layers at four different embryonic points, embryonic day 10 (E10), E13, E16 and E19. Through circRNA differential expression analysis and target miRNA prediction, the circTMTC1 was predicted to participate in the embryonic muscle formation by sponging miRNA, which were verified in vitro experiments.

**Results:** We identified 228 differentially expressed circRNAs between broilers and layers (fold change >2; p-value < 0.05), and 43 circRNAs were differentially expressed at multiple embryonic days. circTMTC1, a novel circRNA transcribed from the TMTC1 gene, was expressed significantly higher in layers than in broilers at E10, E13 and E16. Furthermore, circTMTC1 knockdown accelerated proliferation and differentiation in chicken skeletal muscle satellite cells (SMSCs), besides, circTMTC1-overexpressing cells showed opposite effects. circTMTC1 functioned as a miR-128-3p sponge at the differentiation stage of SMSCs, and circTMTC1 inhibited the expression of miR-128-3p. Furthermore, miR-128-3p promoted differentiation of chicken SMSCs, and circTMTC1 inhibited the promotion effect of miR-128-3p on chicken SMSC differentiation.

**Conclusion:** Our study revealed that circRNAs are differentially expressed during chicken embryonic development between the two chicken models, and circTMTC1 inhibits chicken SMSC differentiation by sponging miR-128-3p.

## Introduction

Muscle plays important roles in initiating movements, supporting respiration and maintaining homeostasis. The functional units of muscle are the muscle fibers, whose number and size determine muscle mass. For most species, the numbers of muscle fibers are established at birth 1. During embryonic myogenesis, mesoderm-derived structures generate the first muscle fibers of the body and additional fibers are generated along these initial template fibers in subsequent waves 2. Myogenesis involves a series of complex cellular and developmental processes, which are regulated by many genes, transcription factors and non-coding RNAs 3-5.

MicroRNAs (miRNAs) represent a class of short non-coding RNAs approximately 22 bp in length that are widely expressed in various cell types and tissues, and exert regulatory roles in biological development. miRNAs have been shown to function as crucial components in the regulatory network for muscle development. Several myogenic miRNAs, such as miR-206 6, miR-1 and miR-133 7, as well as miR-203 8, miR-181 9, and miR-128 10, 11, were demonstrated to regulate muscle development by inhibiting target gene expression.

Circular RNAs (circRNAs), which show a covalently closed loop structure, are generated from back-spliced exons and intron-derived RNAs. The precise biological functions of circRNA are still largely undetermined, but recent studies have shown that some circRNAs that are derived from exons of protein-coding genes can function as miRNA sponges 12. Many differentially expressed circRNAs identified in muscle cells and tissues have been found to regulate miRNA expression by competitive binding. For example, in chicken, circSVIL promotes myogenic differentiation by antagonizing the functions of miR-203 13; circFGFR2 promotes skeletal muscle proliferation and differentiation by sponging miR-133a-5p and miR-29b-1-5p 14; circHIPK3 interacts with miR-30a-3p to regulate myoblast cell development 15; and circRBFOX2s promotes the proliferation of chicken myoblasts by binding miR-1a-3p and miR-206 16. Therefore, while these studies have established a role for some circRNAs, the precise functions and mechanisms of circRNAs in skeletal muscle development are still not completely understood in chicken.

Optimizing skeletal muscle development is of economic importance in farm animals. Notably, after long-term artificial breeding, laying hens and broilers show differences in skeletal muscle development; the skeletal muscle growth rate of broilers far exceeds that of laying hens even under the best feeding conditions, with broilers weighing five times more than laying hens at 6 weeks of age. The comparatively similar genetic backgrounds between broilers and layers enable resources for comparative studies of muscle development.

In this study, we performed RNA-seq in breast muscle of 12 standardized broilers and 12 standardized layers at different embryonic stages to identify specific circRNAs that may function in myogenesis of chicken skeletal muscles.

## Materials and Methods

### Ethics approval

All experimental operations were approved by the Animal Ethics Committee of Sichuan Agricultural University, and the approved number was 2018-177. Relevant guidelines and regulations were followed while performing all the methods.

### Tissue Sample Preparation

The fertilized eggs of Ross 308 and White Longhorn were incubated in the same condition. After sex determination by PCR, only samples identified as male were kept for next experiment. A total of 24 embryonic chickens were used in the study to form eight groups: embryonic day 10 (E10), E13, E16, E19 for Ross 308 and White Longhorn, respectively. Each group included 3 individuals as biological replicates. The breast muscles were collected and immediately frozen in liquid nitrogen, and kept at -80 ℃ until RNA isolation.

### RNA-seq and Data Treatment

Total RNA extraction was performed by Trizol reagent (TaKaRa, Dalian, China). The quantity and quality of RNAs were evaluated by Nanodrop 2000 (Thermo, Waltham, MA, USA) and Agilent 2100 Bioanalyzer (Agilent, Santa Clara, CA, USA), and then DNase (Promega, Madison, WI, USA) were used for remove DNA in the RNA samples. Total RNAs (5 μg) were treated with the epicenter Ribo-ZeroTM Kit (Illumina, San Diego, CA, USA) to remove rRNA for constructing RNA-seq libraries. RNA-seq was performed by Illumina Hiseq 2000 instrument (Illumina) on paired-end module. For raw sequencing data, adapter reads and low-quality reads were removed using Trim Galore software to obtain the clean data. Then clean data reads were mapped to the chicken genome (Gallus_gallus-5.0/galGal5) using BWA software 17. Transcript assembly was performed using Cufflinks software 18.

### Circular RNA identification and Differential expression analysis

Identification of circRNAs was performed using CIRI software 19 and Find_circ software 20. The common prediction results from two software were used as candidates. The expression level of circRNAs were quantified using the number of back-spliced junction reads, and normalized using TPM (transcripts per million) method. Differentially expressed circRNAs among four groups (layer vs. broiler at E10, E13, E16 and E19) were identified using the edgeR software package 21.

### Target miRNA Prediction and circRNA-miRNA Interaction Network Construction

The miRNA target prediction was performed by RNAhybrid software 22 and miRanda software 23. CircRNA-miRNA interaction network construction was performed by Cytoscape software.

### circRNA Verification

The circRNA was validated using PCR with divergent and convergent primers as reported 16. Divergent primer was designed in region about 100 ~ 200bp overlap junction sites, and convergent primer was designed in region of one exon (Supplementary Table. S1). To confirm the junction sequence of circRNA, PCR products of divergent primers were gel purified and submitted for Sanger sequencing at Sangon Biotech Co. Ltd (Shanghai, China).

### Vector Construction and RNA Oligonucleotides

The linear sequence of circTMTC1 was synthesized and cloned into pCD2.1-ciR (Geneseed Biotech, Guangzhou, China) according to the manufacturer's protocol using the KpnI and BamHI (TaKaRa) restriction sites (pCD2.1-circTMTC1). The fragment of the circTMTC1 including binding site of miR-128-3p, was amplified and inserted into Dual luciferase reporter vector (pEZX-FR02) (GeneCopoeia, Rockville, MD, USA) at the 3′ end of Firefly Luciferase gene using restriction enzymes BsiWI and XhoI (TaKaRa) and T4 DNA ligase (pEZX-circTMTC1-WT). The mutant pEZX-circTMTC1-MT was generated by mutating complementary to the seed region of the miR-128-3p using mutagenic primer. pEZX-MSTN-3'UTR-WT vector and pEZX-MSTN-3'UTR-MT vector were constructed using the same method. All constructs were verified by sequencing.

Small interfering RNA (siRNA) overlap junction sites of circTMTC1 and miR-128-3p mimics/inhibitor (Supplementary Table. S2) were synthesized by GenePharma Co. Ltd (Shanghai, China).

### Cell Culture and Treatment

Chicken skeletal muscle satellite cells (SMSCs) from ROSS-308 chicken breast muscle were isolated as described in reports 24, 25. Satellite cells were cultured in growth medium (GM: Dulbecco's modified Eagle medium (DMEM) (Gibco, Langley, OK, USA) + 10% fetal bovine serum (Gibco) + 0.2% penicillin/streptomycin (Invitrogen, Carlsbad, CA, USA) or differentiation medium (DM: Dulbecco's modified Eagle medium + 2% horse serum (Gibco, USA)) at 37℃ in a 5% CO2 humidified atmosphere. DF-1 cells were also cultured in DMEM with 10% fetal bovine serum.

Satellite cells were transfected with miR-128-3p mimics, inhibitor, si-circTMTC1 or pcD-circTMTC1 using Lipofectamine 3000 (Invitrogen) according to the manufacturer's instructions. Cells were transfected when cell confluence reached approximately 90% and cultured in DM to study cell differentiation, while study cell proliferation was transfected at 50% and cultured in GM.

### RNA Isolation, cDNA Synthesis, and Real-Time qRT-PCR

Total RNA isolation was performed by Trizol reagent. cDNA synthesis was performed by TransScript One-Step gDNA Removal and cDNA Synthesis SuperMix (TransGen, Beijing, China) and One Step miRNA cDNA Synthesis Kit (HaiGene, Haerbin, China). For RNase R treatment, 1 μg of total RNA was incubated for 10 min at 37 ℃ with 1 unit of RNase R, and then deactivated RNase R at 90℃ for 10 min. The qRT-PCR analyses were performed using TB Green PCR Master Mix (Takara). For each time-point, qRT-PCR was done on three biological replicates. U6 (for miRNA) and β-actin gene was used as internal control. The primers are listed in Supplementary Table. S3. The 2^-ΔΔCt^ method was used to analyze the relative expression level of qRT-PCR data.

### Luciferase Reporter Assay

For luciferase reporter assay, DF-1 cells were seeded in 48-well plates and co-transfected with wild type (WT) or mutated (MT) reporter vector and miR-128-3p mimics or negative mimic. After transfected for 48 h, the luminescent values of firefly and Renilla luciferase were detected using Dual-GLO Luciferase Assay System Kit (Promega) with a Fluorescence/ Multi-Detection Microplate Reader (Biotek, Shoreline, WA, USA).

### CCK-8 Assay and EdU Assay

For the CCK-8 assay, SMSCs were plated into 96-well culture plates at a density of 1 × 10^4^ cells/well in 100 μL of GM per well, and each treatment group had eight independent replicates. After transfection for 12 h, 24 h, 48 h and 72 h, 10 μL of CCK-8 reagent (Multisciences, Hangzhou, China) was added to each well and incubated at 37 ºC for 3 h. The absorbance of each sample at 450 nm wavelength was detected using a microplate reader.

For the EdU assay, SMSCs were plated into 48-well culture plates and cultured in GM, each treatment group had three independent replicates. After transfection for 48 h, EdU assays were performed by the Cell-Light EdU DNA cell proliferation kit (RiboBio, Guangzhou, China) according to the manufacturer's instructions.

### Flow Cytometry Analysis of Cell Cycle

SMSCs were seeded in 6-well plates and cultured in GM. After transfection for 48 h, cells were collected and suspended in 75% ethanol overnight at -20 ℃. Then, the cells were collected and incubated with 500 μl PI/RNase Staining Buffer Solution (BD Biosciences, Franklin Lakes, USA). Analyses were performed using a BD AccuriC6 flow cytometer (BD Biosciences) and Modfit software.

### Biotin-Coupled miRNA RNA Pull Down Assay

The method of biotin-coupled miRNA pull down assay reference to previous reports 14, 26. Briefly, SMSCs were cultured in T75 cell culture bottle and co-transfected with 50 nM of 3′ end biotinylated miR-128-3p mimic or negative mimic and 20 μg pCD2.1-circTMTC1 vector. After 24 h of transfection, cells were collected and lysed in lysis buffer, 100 μl washed streptavidin magnetic beads were blocked for 2 h and then divided into several centrifuge tubes and incubated with cell lysate for 5 h on a mini tube rotator at a low speed. At last, beads were washed and collected in 1 mL Trizol reagent waiting for RNA isolation.

### Nucleus and Cytoplasm Separation Assay

We performed nucleus and cytoplasm separation assay using NE-PER™ Nuclear and Cytoplasmic Extraction Reagents (Thermo Fisher) according to the manufacturer's instructions.

### Western Blot Assay

SMSCs were seeded in 6-well plates and cultured in DM. After transfection for 72 h, cells were collected and proteins were extracted using lysis buffer and the concentration determined by a bicinchoninic acid (BCA) protein assay kit (Beyotime, Shanghai, China). Total proteins (30 μg) were separated on a 12% SDS-PAGE, and transferred to a 0.2 mm polyvinylidene fluoride (PVDF) membrane that was soaked in formaldehyde and then blocked with 5% skim milk in Tris saline with Tween (TBST) buffer for about 2h at room temperature. The membrane was then incubated overnight with primary antibodies specific for anti-Myosin (Santa Cruz Biotechnology, Santa Cruz, CA, USA; 1:500), anti-MYOG (Santa Cruz Biotechnology; 1:500), and anti-β-actin (Santa Cruz Biotechnology; 1:1000) at 4 ℃. The PVDF membrane was washed three times with TBST buffer and then incubated with secondary antibody for 2 h at room temperature. β-actin was used as the internal control with a secondary antibody that was horseradish peroxidase (HRP)-labeled anti-mouse immunoglobulin G (IgG) (ZenBio, Beijing, China; 1:2000). Finally, antibody reacting bands were detected using enhanced chemiluminescence (ECL) luminous fluid (Solarbio, Beijing, China).

### Immunofluorescence Assay

SMSCs were seeded in 24-well plates and cultured in DM. After transfection for 72 h, cells were fixed in 4% formaldehyde for 30 min then washed three times with PBS for 5 min. Subsequently, the cells were permeabilized by adding 0.1% Triton X-100 for 20 min and blocked with 5% goat serum (Beyotime) for 30 min. After incubation with Myosin (Santa Cruz, USA; 1:200) at 4℃ for 12 h, the Rhodamine (TRITC) AffiniPure Goat Anti-Mouse IgG (ZenBio; 1:200) was added and the cells were incubated at 37℃ for 1 h. The cell nuclei were stained with DAPI (Beyotime; 1:50) for 5 min. Images were obtained with a fluorescence microscope (Olympus, Japan).

### Statistical Analysis

Data are presented as least squares means ± standard error of the mean (SEM). For two group comparison analysis, statistical significance of differences between means was analyzed by unpaired Student's t-test. For multiple comparison analysis, data were analyzed by one-way ANOVA analyse using SPSS 20.0 (SPSS Inc., USA), and values were considered statistically different at *p* < 0.05.

## Results

### Identification of CircRNAs from RNA-seq

**Figure 1:**
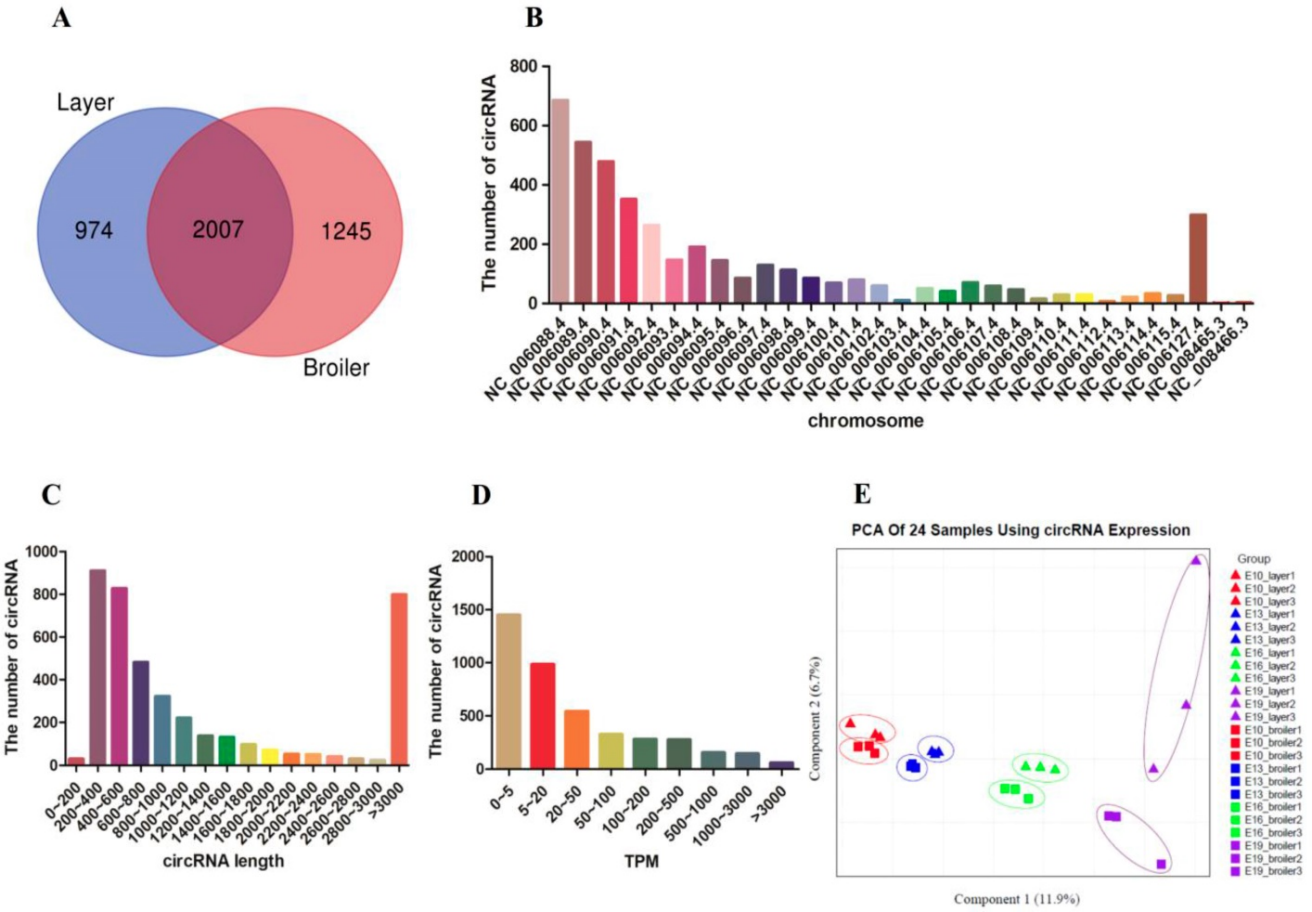
** Identification and description of circRNAs in breast muscle of chickens. (A)** Venn diagrams of circRNAs in embryonic muscle oftwo chicken lines. **(B)** Distribution of total circRNAs in different chicken chromosomes. **(C)** Length size distribution of total circRNAs. **(D)** Expression levels distribution of total circRNAs. **(E)** The PCA plot of 24 samples using all circRNA expression.

We performed RNA-seq in breast muscle of 12 broilers and 12 layers and approximately 3 billion reads were generated, with each sample yielding more than 100 million reads. After removing adapters and reads with low quality, the clean data were mapped to the chicken reference genome (Gallus gallus-5.0/galGal5) (Supplementary Table. S4). CIRI and Find_circ software were used for circRNA identification, and the identified transcripts that satisfied the criteria of both software were regarded as potential circRNAs. Approximately 592~1629 different types of circRNAs were identified in each sample (Supplementary Table. S5). A total of 4226 circRNAs were identified from the 24 samples (Supplementary Table. S6); among them, 2981 were identified in layers, 3252 were identified in broilers, and 2007 were identified in both layers and broilers (Fig. 1A). We found the genomic loci where circRNAs transcript from to be widely distributed on all chromosomes, and there was a general trend that numbers of circRNAs per chromosome increased with absolute chromosome length (Fig. 1B). The length of the circRNAs ranged from 42 to 98004 nucleotides (nt) and the mean length was 731 nt; more than 60% of the circRNAs showed a length of less than 1000 nt (Fig. 1C). The circRNAs showed a wide range of expression; most circRNAs showed a low expression level (mean TPM < 200, n=3585), whereas few circRNAs were highly expressed (mean TPM > 3000, n=60) (Fig. 1D). Principal component analysis was performed using all circRNA expressions from 24 samples, and the result showed clear classification among different groups, which indicated the good quality of our samples (Fig. 1E).

### Analysis of CircRNAs Differentially Expressed between Broilers and Layers

**Figure 2:**
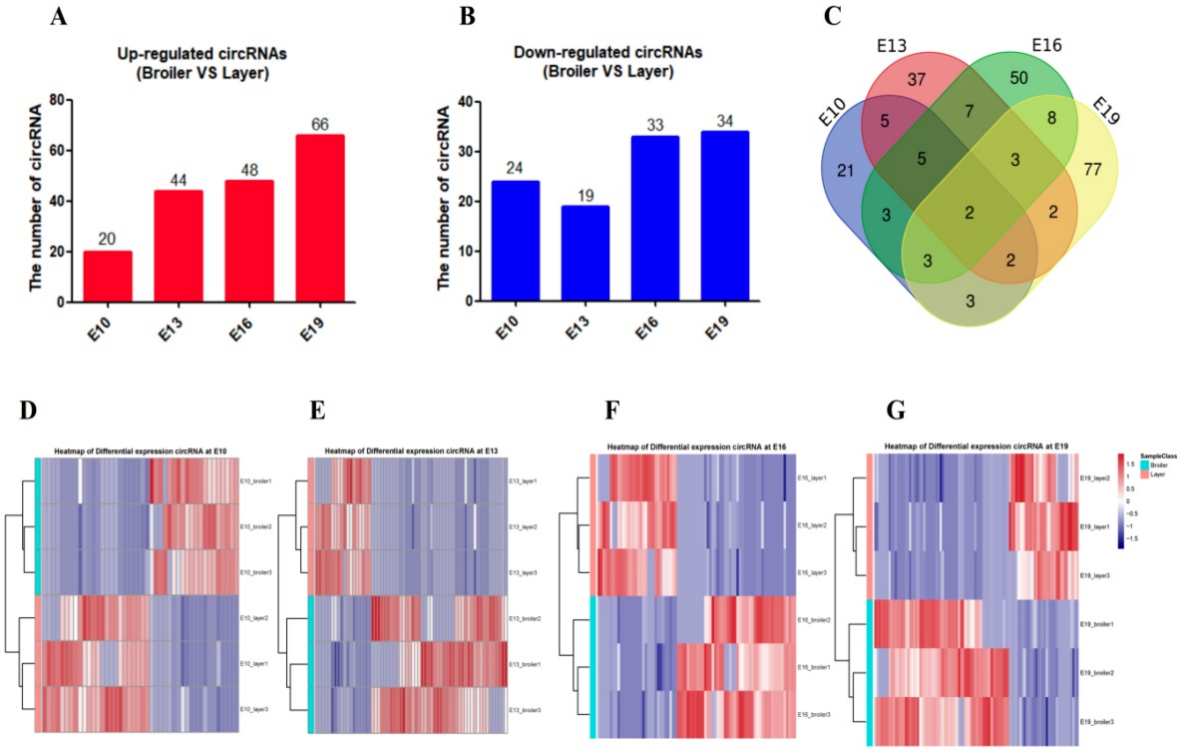
** Description of differentially expressed circRNAs between broilers and layers**. **(A, B)** Up-regulated and down-regulated circRNA numbers in each comparison group (Broilers vs. Layers). **(C)** Venn diagrams of differentially expressed circRNAs in four comparison group (n=228; *P* < 0.05, fold change ≥ 2). **(D-G)** Heatmap of differentially expressed circRNAs in each paired group (Broilers vs. Layers).

Differentially expressed circRNA (DEC) calling was performed between the two chicken lines at each of the four time points (E10, E13, E16, and E19). DECs were identified using two criteria: fold change (FC) ≥ 2.0 and *P* < 0.05. A total of 228 DECs were detected between broilers and layers at the four time points: 44 in the E10 dataset (20 up-regulated, 24 down-regulated), 62 in the E13 dataset (44 up-regulated, 19 down-regulated), 80 in the E16 dataset (48 up-regulated, 33 down-regulated), and 100 in the E19 dataset (66 up-regulated, 34 down-regulated) (Fig. 2A and B, Supplementary Table. S7). Most DECs (n=185) were only present at one time point, while fewer circRNAs (n=43) were identified at multiple embryonic days (Fig. 2C). Clustering analysis indicated that broilers and layers have different circRNA expression patterns (Fig. 2D-G). We selected the top 10 highly expressed DECs as candidates for biological function verification (Table. 1).

### Construction of the CircRNA-miRNA Interaction Network

**Figure 3:**
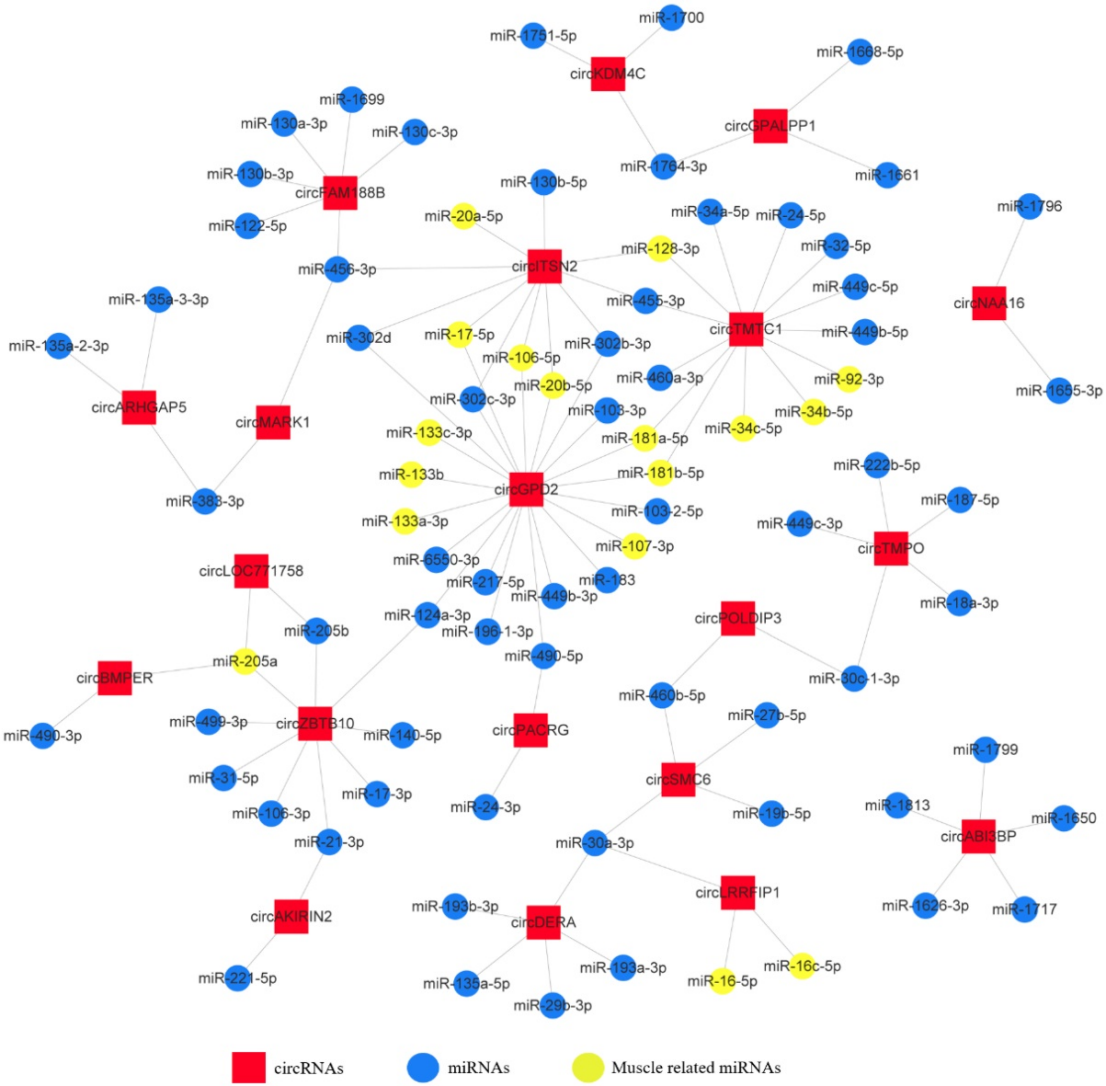
The circRNA-microRNA interaction network map of the 20 most abundant differentially expressed exonic circRNAs with their potential target miRNAs.

To investigate the functions of circRNAs during embryonic chicken muscle development, we constructed the circRNA-microRNA interaction network based on their predicted relationship. The miRNA target prediction of differentially expressed exonic circRNAs was performed by RNAhybrid and miRanda software; the targets that were predicted by both software were selected as candidates (Supplementary Table. S8). The 20 most abundant differentially expressed exonic circRNAs with their potential target miRNAs are shown in the circRNA-miRNA interaction network map (Fig. 3). The results showed that some circRNAs associated with several muscle-related miRNAs such as miR-133, miR-181, miR-128 and miR-17~92, which suggested these circRNAs, may have potential roles in chicken muscle development.

### Experimental Validation of DECs

**Figure 4:**
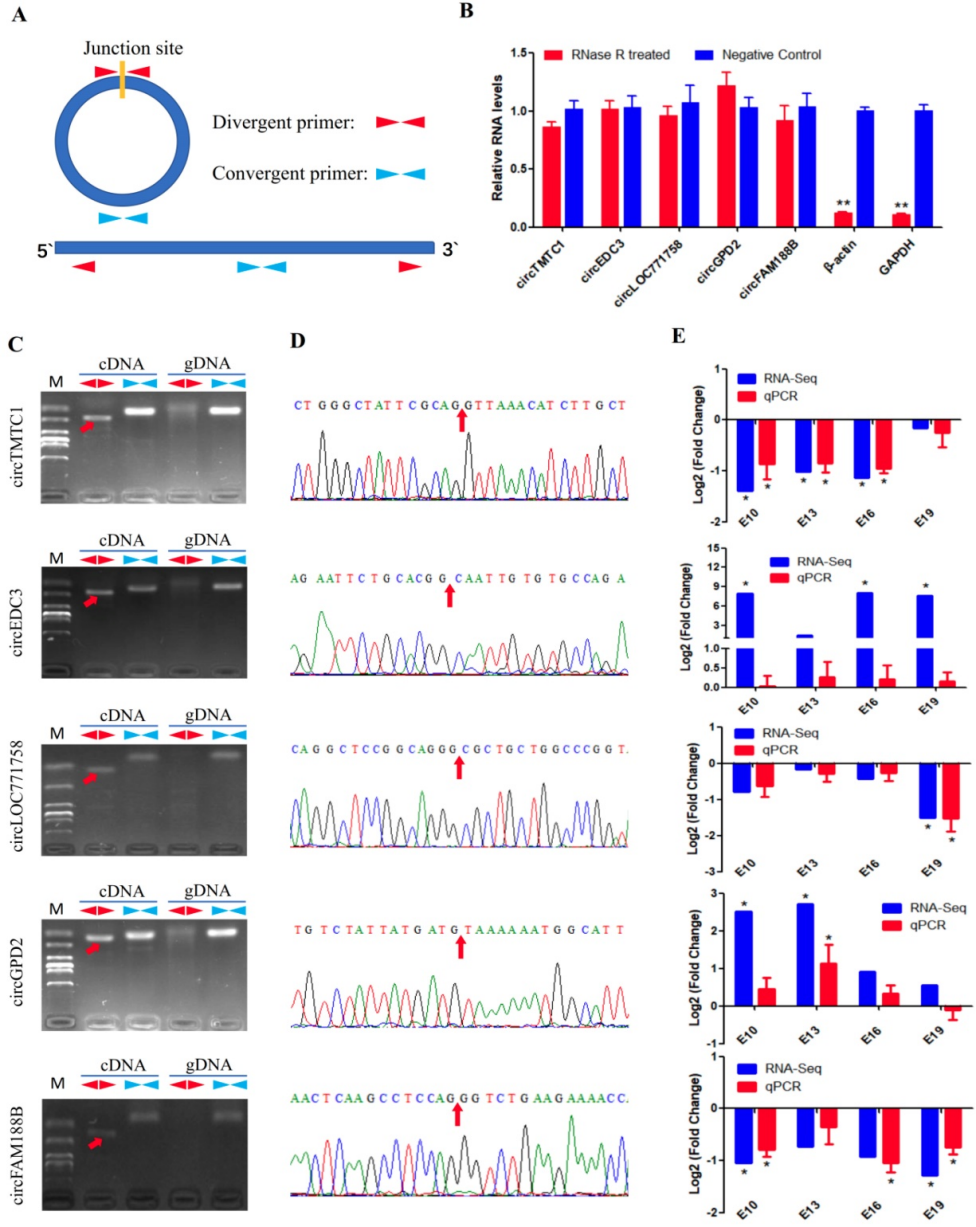
** Experimental validation of differentially expressed circRNAs**. **(A)** The schematic diagram of divergent primers and convergent primers. **(B)** qRT-PCR results showing resistance of circRNAs and reference genes to RNaseR digestion. **(C)** Divergent primers and convergent primers amplify results of circRNAs in cDNA and gDNA samples. **(D)** Sanger sequencing confirmed the back-splicing junction sequence of circRNAs (Red arrow points to the splicing site). **(E)** qRT-PCR validation of five differentially expressed circRNAs in four comparison groups. Data are presented as means ± S.E.M. for three individuals. The Student's t-test was used to compare expression levels among different groups. *P < 0.05; **P < 0.01.

We next validated the five differentially expressed circRNAs identified from sequencing data using different primers (Fig. 4A). RNase R was used to confirm the circularity of circRNAs. We treated total RNAs with RNase R treatment and performed qRT-PCR; the results showed that the circRNAs were more resistant to RNase R than GAPDH and β-actin mRNA (Fig. 4B). Divergent primers from five circRNAs produced a single distinct band only in cDNA samples (Fig. 4C), indicating these circRNAs are back-splicing products from the chicken genome. The PCR products from divergent primers were sequenced for junction site verification (Fig. 4D). We also examined the expression of the five DECs in E10, E13, E16 and E19 comparison groups by qRT-PCR. The expression patterns of these circRNAs, except circEDC3, were almost consistent with the RNA-Seq results (Fig. 4E), suggesting a reliable circRNA-Seq outcome.

### CircRNA circTMTC1 is Differentially Expressed during Chicken Breast Muscle Development

**Figure 5:**
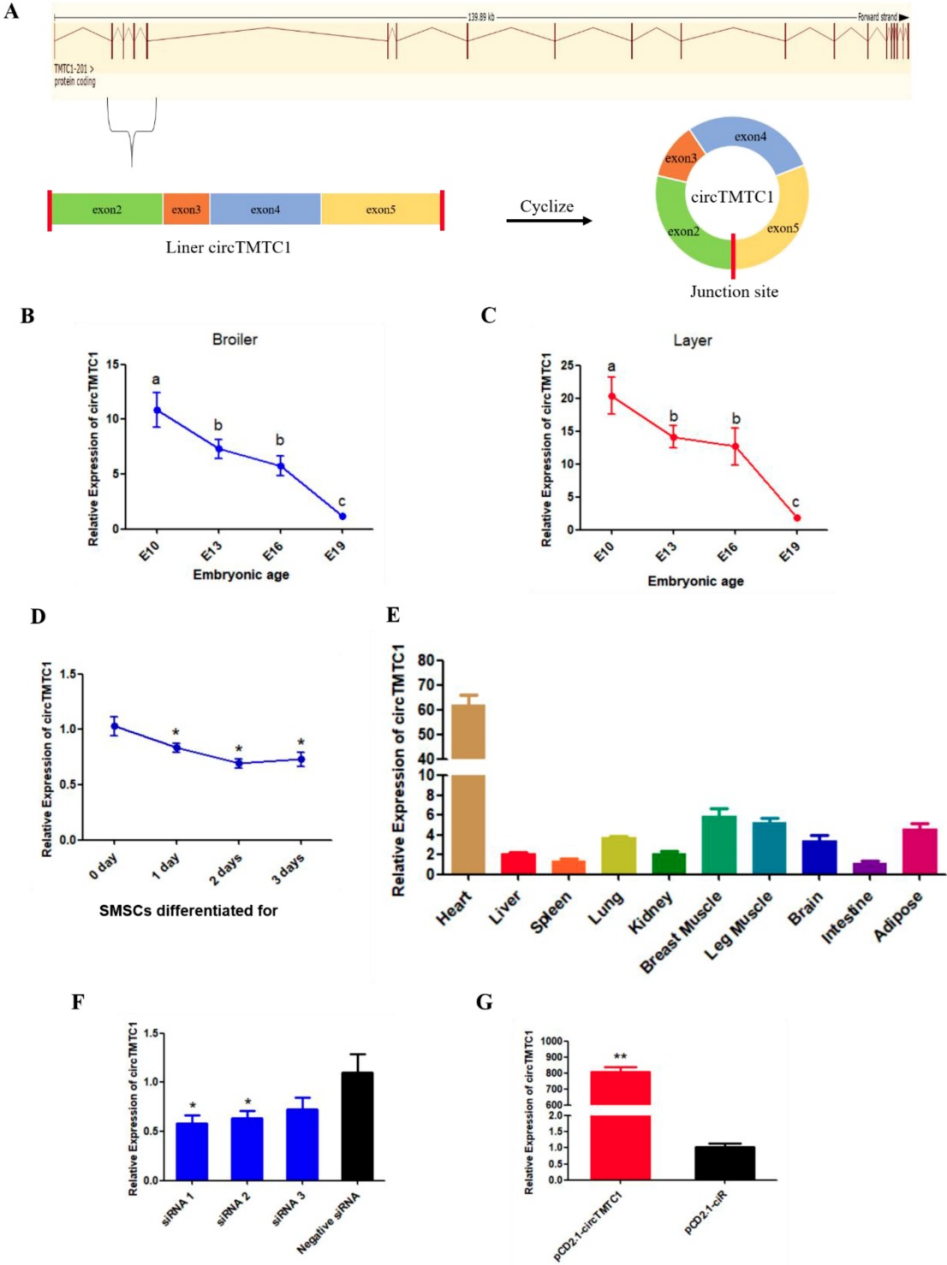
** The source and expression pattern of circTMTC1**. **(A)** Schematic diagram of circTMTC1 derived from TMTC1 gene. **(B)** The expression levels of circTMTC1 in broilers breast muscle at four embryonic ages. **(C)** The expression levels of circTMTC1 in layers breast muscle at four embryonic ages. **(D)** The expression levels of circTMTC1 during chicken skeletal muscle satellite cells differentiation was detected by qRT-PCR. **(E)** Expression levels of circTMTC1 in different tissues of 30-day-old chicken. **(F, G)** The expression levels of circTMTC1 were detected by qRT-PCR in SMSCs which transfected with siRNA of circTMTC1 or negative siRNA, pCD2.1-circTMTC1 or pCD2.1-ciR. The One-way ANOVA **(B, C)** and Student's t-test **(D, F and G)** were used to compare expression levels among different groups. *P < 0.05; **P < 0.01; ^a,b^ *P* < 0.05.

We found that circTMTC1 was highly expressed in embryonic chicken breast muscle (ranking of expression level: 58/4226) and differentially expressed in layers and broilers at E10, E13 and E16 (*P* < 0.05; Fig. 4E). circTMTC1 is derived from exon 2 to 5 of the TMTC1 gene on chicken chromosome 1 (Fig. 5A). To examine the relationship between circTMTC1 and muscle development, we measured its expression in breast muscle during chicken embryonic development and found that its expression was significantly decreased from E10 to E19 both in broiler (*P* < 0.05; Fig. 5B) and layer (*P* < 0.05; Fig. 5C). Furthermore, the expression of circTMTC1 was higher at the proliferation period compared with the differentiation period and was decreased during skeletal muscle satellite cell (SMSC) differentiation (*P* < 0.05; Fig. 5D). We also found that circTMTC1 was enriched in heart and highly expressed in skeletal muscles (Fig. 5E).

### CircTMTC1 Inhibits Proliferation of Chicken SMSCs

We next evaluated the function of circTMTC1 by modulating its expression using siRNA targeting circTMTC1 or an overexpression vector expressing circTMTC1. We used three circTMTC1 siRNAs and confirmed that circTMTC1 was significantly decreased by transfection with all three siRNAs compared with control siRNA (*P* < 0.05; Fig. 5F). Among the three siRNAs, siRNA-1 showed the most effective knockdown effects and was thus chosen for subsequent experiments. Furthermore, circTMTC1 level in cells transfected with the overexpression vector was over 800 times higher than cells transfected with empty vector (*P* < 0.01; Fig. 5G and Supplementary Fig. S1).

**Figure 6:**
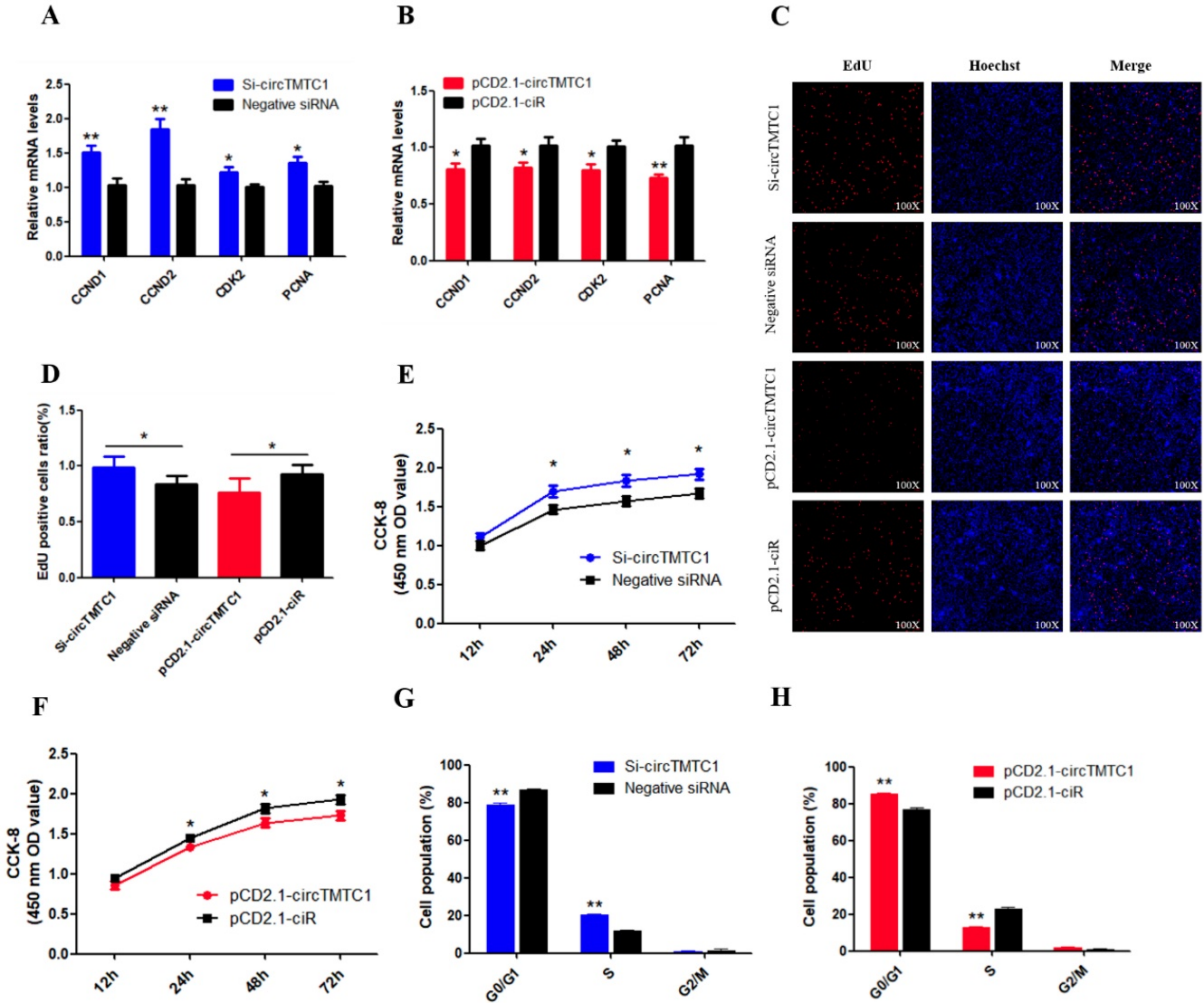
** Chicken circTMTC1 inhibits the proliferation of skeletal muscle satellite cells. (A)** The mRNA levels of cell proliferation related genes were detected by qRT-PCR in SMSCs which transfected with circTMTC1 siRNA or negative siRNA. **(B)** The mRNA levels of cell proliferation related genes were detected by qRT-PCR in SMSCs which transfected with pCD2.1-circTMTC1 or pCD2.1-ciR. **(C)** EdU assays for SMSCs transfected with siRNAs or vectors. EdU (red) fluorescence indicates proliferation. Nuclei are indicated by Hoechst (blue) fluorescence. All photomicrographs are at 100 × magnification. **(D)** The percentage of EdU positive cells per total cell numbers. **(E)** CCK-8 assays for SMSCs transfected with siRNAs or vectors. **(F)** Flow Cytometry of cell cycle analysis for SMSCs transfected with circTMTC1 siRNA or negative siRNA. **(G)** Flow Cytometry of cell cycle analysis for SMSCs transfected with pCD2.1-circTMTC1 or pCD2.1-ciR vector. Data are presented as means ±S.E.M. for three individuals. The Student's t-test was used to compare the data among different groups. *P < 0.05; **P < 0.01.

To determine the biological function of cirTMTC1 in SMSCs, we next examined the effect of circTMTC1 on the mRNA expression levels of cell proliferation-related genes cyclin D1 (CCND1), cyclin D2 (CCND2), cyclin-dependent-kinase 2 (CDK2), and proliferating cell nuclear antigen (PCNA). Knockdown of circTMTC1 promoted CCND1, CCND2, CDK2, and PCNA mRNA expressions (Fig. 6A), and these genes were down-regulated by circTMTC1 overexpression (Fig. 6B). We also examined proliferation by EdU assay and found that the ratio of EdU-positive cells was increased by circTMTC1 knockdown and decreased by circTMTC1 overexpression (Fig. 6C and D). CCK-8 assays showed similar results with EdU assay (Fig. 6E). In addition, cell cycle analysis revealed that knocking down circTMTC1 increased the number of SMSCs in S phase and decreased the proportion of cells in G1/G0 phase (*P* < 0.01; Fig. 6F), whereas overexpression of circTMTC1 reduced the number of S phase cells and increased the population of G0/G1 cells (*P* < 0.01; Fig. 6G and Supplementary Fig. S2). Together these results suggested that circTMTC1 inhibits SMSC proliferation.

### CircTMTC1 Represses Differentiation of Chicken SMSCs

**Figure 7:**
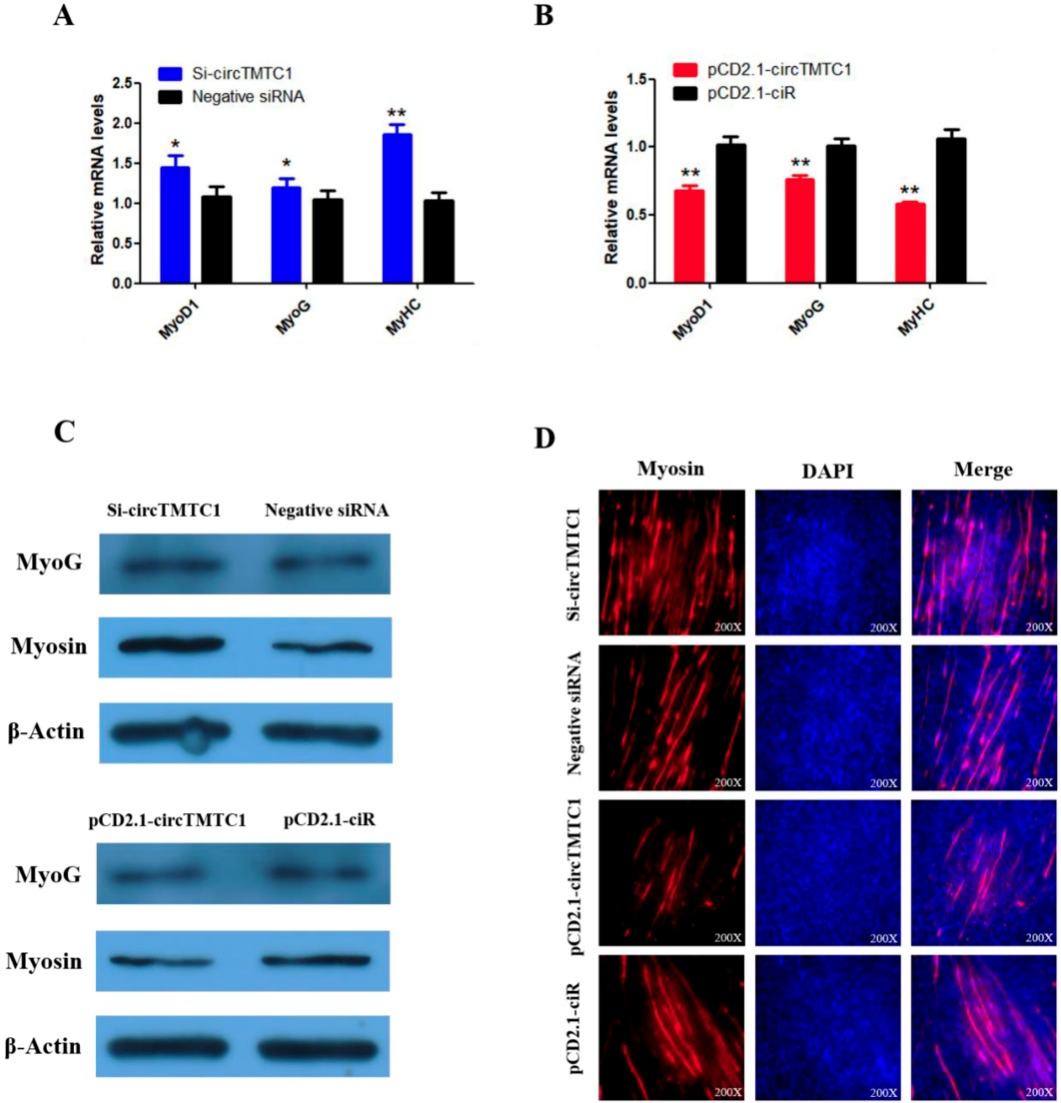
** Chicken circTMTC1 inhibits the differentiation of skeletal muscle satellite cells**. **(A, B)** The mRNA levels of marker genes for muscle cells differentiation were detected by qRT-PCR in SMSCs which transfected with circTMTC1 siRNA or negative siRNA, pCD2.1-circTMTC1 or pCD2.1-ciR. **(C)** The expression of MYOG and Myosin was determined by Western blot in SMSCs which transfected with circTMTC1 siRNA or negative siRNA, pCD2.1-circTMTC1 or pCD2.1-ciR. **(D)** Immunofluorescence analysis of Myosin-staining cells after knock down or over expression of circTMTC1. Data are presented as means ±S.E.M. for three individuals. The Student's t-test was used to compare expression levels among different groups. *P < 0.05; **P < 0.01.

To explore whether circTMTC1 regulates SMSC differentiation, we examined the expressions of three muscle differentiation marker genes, myoblast determination protein 1 (MyoD1), myogenin (MyoG) and myosin heavy chain (MyHC), by qRT-PCR. The results showed that circTMTC1 knockdown increased the mRNA abundances of all three markers (*P* < 0.05; Fig. 7A), while the mRNA expression levels were decreased by circTMTC1 overexpression (*P* < 0.01; Fig. 7B). Similarly, MYOG and Myosin protein levels increased after knockdown of circTMTC1 but decreased when circTMTC1 was overexpressed (Fig. 7C). Furthermore, immunofluorescence of myosin revealed that knockdown of circTMTC1 promoted myotube formation, whereas overexpression of circTMTC1 inhibited myotube formation (Fig. 7D).

### CircTMTC1 Acts as a Competing Endogenous RNA for miR-128-3p

**Figure 8:**
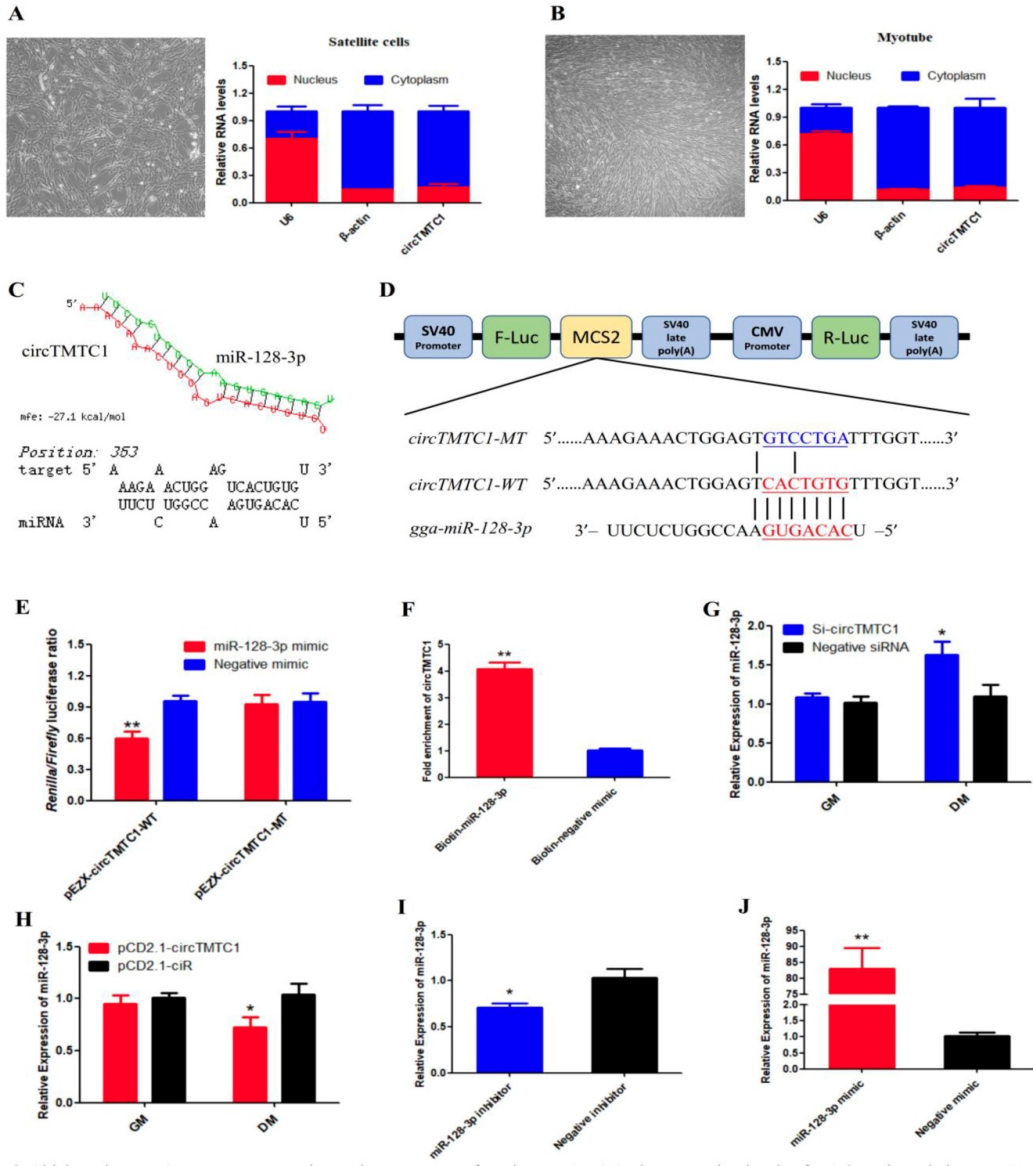
** Chicken circTMTC1 acts as a competing endogenous RNA for miR-128-3p. (A)** The expression levels of U6, β-Actin and circTMTC1 in nuclei (red) and cytoplasm (blue) in undifferentiated SMSCs. **(B)** The expression levels of U6, β-Actin and circTMTC1 in nuclei and cytoplasm in differentiated myotubes. **(C)** miR-128-3p targeting site in circTMTC1 analysed by RNAhybrid software. **(D)** Schematic diagram of luciferase reporter (pEZX-FR02) contain wild type or mutant type of miR-128-3p targeting site. **(E)** Ratio of Firefly luciferase to Renilla luciferase activity of DF-1 cells after co-transfected with pEZX-circTMTC1-WT/pEZX-circTMTC1-MT and miR-128-3p mimic/negative mimic. **(F)** RNA pull-down from the SMSCs after transfection with 3′ end biotinylated miR-128-3p, or negative mimic control. **(G)** The expression levels of miR-128-3p were detected by qRT-PCR in SMSCs which transfected with circTMTC1 siRNA or negative siRNA. **(H)** The expression levels of miR-128-3p were detected by qRT-PCR in SMSCs which transfected with pCD2.1-circTMTC1 or pCD2.1-ciR. **(I, J)** The expression levels of miR-128-3p were detected by qRT-PCR in SMSCs which transfected with miR-128-3p inhibitor or negative inhibitor, miR-128-3p mimic or negative mimic. Data are presented as means ± S.E.M. for three individuals. The Student's t-test was used to compare expression levels among different groups. *P < 0.05; **P < 0.01.

To determine the mechanism by which circTMTC1 inhibits SMSC proliferation and differentiation, we collected undifferentiated cells and differentiated myotubes and separated nuclei and cytoplasm for qRT-PCR. circTMTC1 was mainly located in the cytoplasm (Fig. 8A and B), suggesting it may play a role in post-transcriptional regulation. Previous studies have shown that circRNAs located in cytoplasm can function as miRNA sponges^27^, ^28^. Thus, we next investigated the possibly that circTMTC1 may interact with miRNAs. RNAhybrid software prediction results indicated that circTMTC1 contains a potential binding site for miR-128-3p (Fig. 8C).

To determine whether circTMTC1 interacts with miR-128-3p, we constructed a dual-luciferase reporter with the wild-type linear sequence of circTMTC1 at the 3′ end of the firefly luciferase gene or a mutant in which the miR-128-3p binding site was mutated (Fig. 8D). Luciferase activity of the wild-type plasmid was significantly reduced by co-transfection with the miR-128-3p mimic compared with the control mimic (*P* < 0.05), but the mutant reporter showed no response to the miR-128-3p mimic (*P* > 0.05; Fig. 8E).

To confirm the interaction between circTMTC1 and miR-128-3p, RNA pull down assay was performed in SMSCs transfected with miR-128-3p biotinylated at the 3′ end or negative mimic control. Significantly greater amounts of circTMTC1 were captured by biotinylated-miR-128-3p compared with control mimic (*P* < 0.01; Fig. 8F). Moreover, knockdown of circTMTC1 significantly increased miR-128-3p expression at the differentiation stage of SMSCs (P < 0.05; Fig. 8G), while overexpressed circTMTC1 significantly decreased the level of miR-128-3p at the differentiation stage (*P* < 0.05; Fig. 8H), but not in the proliferation stage. Together, these findings indicate that circTMTC1 can sponge miR-128-3p in the differentiation period of SMSCs.

### miR-128-3p Promotes Differentiation of Chicken SMSCs

To explore the function of miR-128-3p in chicken SMSCs, we modulated miR-128-3p expression using miR-128-3p mimic or inhibitor. We confirmed that miR-128-3p levels were significantly decreased in SMSCs by the miR-128-3p inhibitor (*P* < 0.05; Fig. 8I) and increased by more than 80-fold in SMSCs with the miR-128-3p mimic (*P* < 0.01; Fig. 8J).

**Figure 9:**
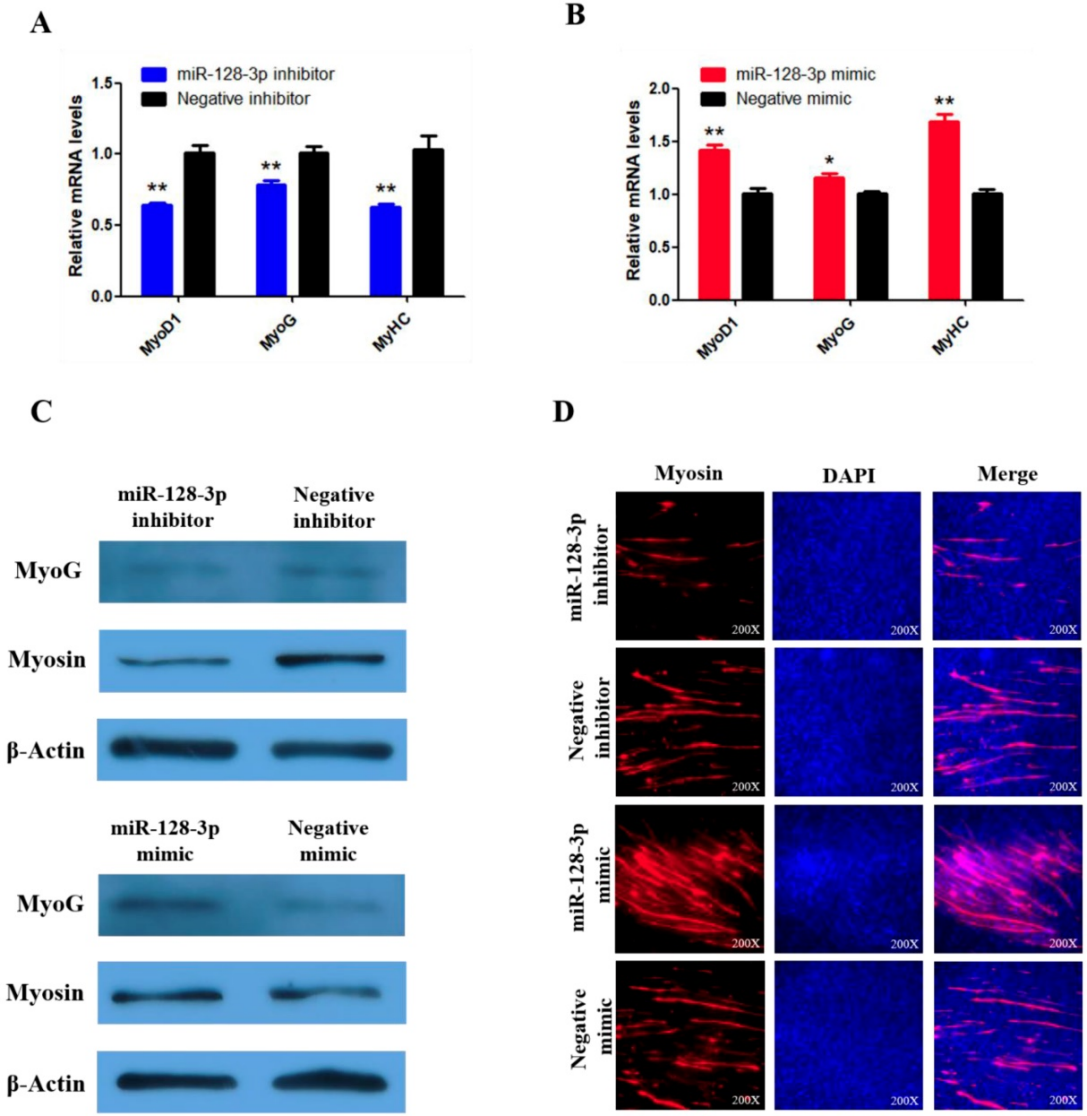
** miR-128-3p promotes the differentiation of SMSCs in chicken. (A, B)** The mRNA levels of markers for muscle cells differentiation were detected by qRT-PCR in SMSCs which transfected with miR-128-3p inhibitor or negative inhibitor, miR-128-3p mimic or negative mimic.** (C)** The expression of MyoG and Myosin was determined by Western blot in SMSCs which transfected with miR-128-3p inhibitor or negative inhibitor, miR-128-3p mimic or negative mimic. **(D)** Immunofluorescence analysis of Myosin-staining cells after knock down or over expression of miR-128-3p. Data are presented as means ± S.E.M. for three individuals. The Student's t-test was used to compare expression among different groups. *P < 0.05; **P < 0.01.

To explore whether miR-128-3p regulates SMSC differentiation, we examined the expression of three established muscle differentiation marker genes MyoG, MyoD1 and MyHC. The mRNA abundances of MyoG, MyoD1, and MyHC were reduced by miR-128-3p inhibitor compared with the negative control (*P* < 0.01; Fig. 9A) and increased in response to the miR-128-3p mimic (Fig. 9B). MyoG and Myosin protein levels reflected the mRNA results (Fig. 9C). Furthermore, myosin immunofluorescence revealed that knockdown of miR-128-3p inhibited myotube formation, while overexpression of miR-128-3p promoted myotube formation (Fig. 9D). Together, these results indicate that miR-128-3p promotes chicken SMSC differentiation.

### CircTMTC1 Inhibits the Effect of miR-128-3p on Promoting SMSCs Differentiation

**Figure 10:**
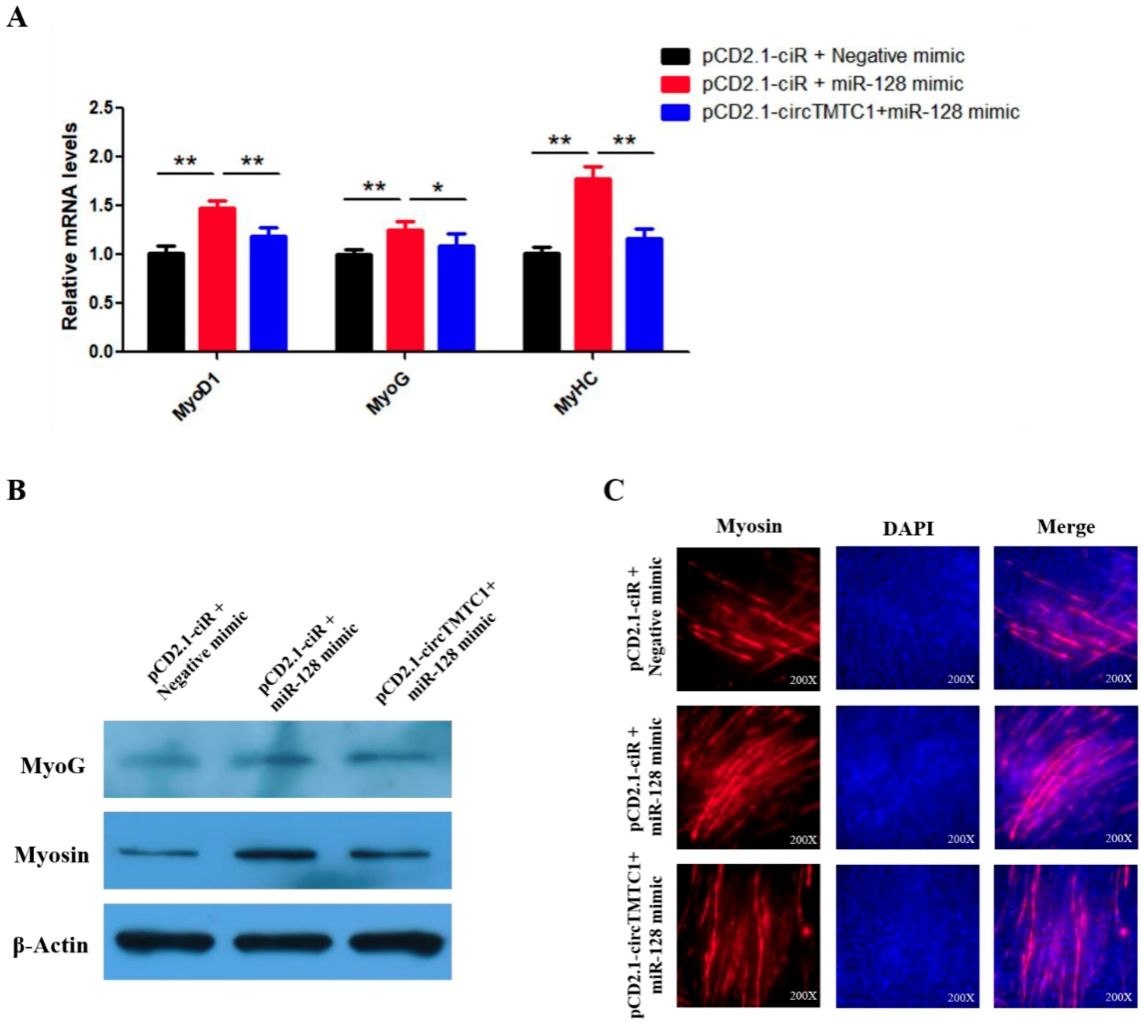
** CircTMTC1 eliminates the promotion effect of miR-128-3p on SMSCs differentiation. (A)** The mRNA levels of marker genes for muscle cells differentiation were detected by qRT-PCR in SMSCs after co-transfected with vectors and mimics.** (B)** The protein levels of marker genes for muscle cells differentiation were detected by qRT-PCR in SMSCs after co-transfected with vectors and mimics. **(C)** Immunofluorescence analysis of Myosin-staining cells after co-transfected with vectors and mimics. Data are presented as means ± S.E.M. for three individuals. The Student's t-test was used to compare the data among different groups. *P < 0.05; **P < 0.01.

Our results suggest that circTMTC1 functions by binding and inhibiting miR-128-3p in the differentiation SMSCs. Thus, we next performed rescue experiments to assess whether the effect of miR-128-3p on promoting SMSC differentiation could be blocked by circTMTC1 overexpression. Indeed, qRT-PCR and western blot showed that circTMTC1 overexpression could block the ability of miR-128-3p to stimulate both mRNA (*P* < 0.05; Fig. 10A) and protein levels (Fig. 10B) of muscle differentiation marker genes. Myosin immunofluorescence further showed that circTMTC1 could block the positive effect of miR-128-3p on myotube formation (Fig. 10C).

### MSTN Is a Target Gene of miR-128-3p and CircTMTC1 Can Relieve the Inhibition of miR-128-3p on MSTN

**Figure 11:**
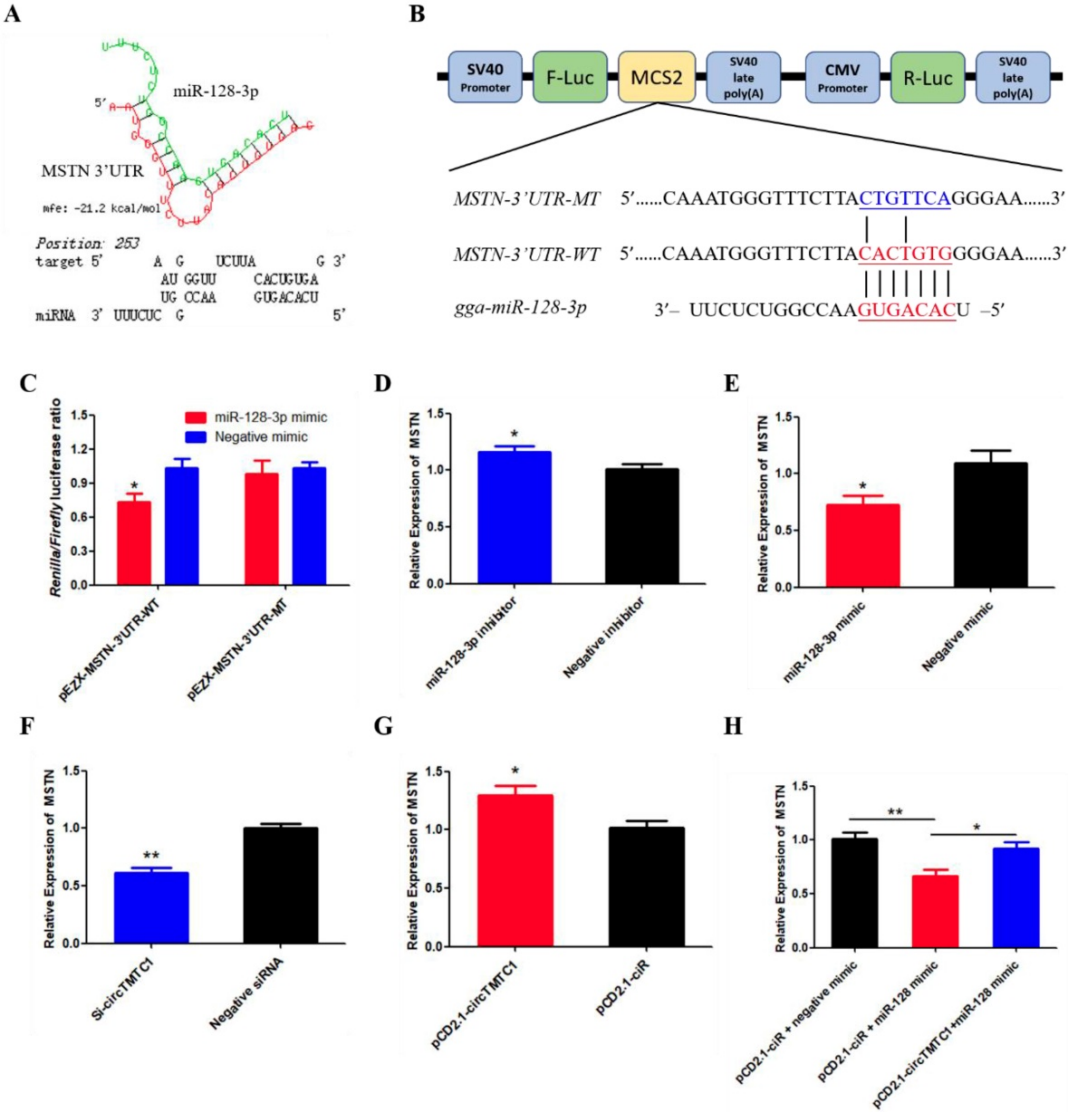
** MSTN is a target gene of miR-128-3p and circTMTC1 can relieve the inhibition of miR-128-3p on MSTN**. **(A)** miR-128-3p targeting site in MSTN-3'UTR analysed by RNAhybrid software. **(B)** Schematic diagram of luciferase reporter (pEZX-FR02) contain wild type or mutant type of miR-128-3p targeting site. **(C)** Ratio of Firefly luciferase to Renilla luciferase activity of DF-1 cells after co-transfected with pEZX- MSTN-3'UTR -WT/pEZX- MSTN-3'UTR -MT and miR-128-3p mimic/negative mimic. **(D, E)** The expression levels of MSTN were detected by qRT-PCR in SMSCs which transfected with miR-128-3p inhibitor or negative inhibitor, miR-128-3p mimic or negative mimic. **(F, G)** The expression levels of MSTN were detected by qRT-PCR in SMSCs which transfected with siRNA of circTMTC1 or negative siRNA, pCD2.1-circTMTC1 or pCD2.1-ciR. **(H)** The mRNA levels of MSTN were detected by qRT-PCR in SMSCs which co-transfected with vectors and mimics. Data are presented as means ± S.E.M. for three individuals. The Student's t-test was used to compare the data among different groups. *P < 0.05; **P < 0.01.

To determine which gene targeted by miR-128-3p to promotes SMSC differentiation. The TargetScan website were used and we found that Myostatin (MSTN) is the most attractive candidate because it has well-established roles in chicken SMSCs differentiation. Furthermore, RNAhybrid software predicted results showed the binding site of miR-128-3p and MSTN 3'UTR (Fig. 11A). The dual-luciferase reporter assay verified that miR-128-3p could combined with the site of wild type reporter, but not the mutant type reporter (*P* < 0.05; Fig. 11B and C). In addition, knockdown of miR-128-3p significantly increased MSTN expression (P < 0.05; Fig. 11D), while overexpressed miR-128-3p significantly decreased the level of MSTN (*P* < 0.05; Fig. 11E). We also explored whether circTMTC1 regulates the expression of MSTN. The mRNA level of MSTN was reduced by circTMTC1 siRNA (*P* < 0.01; Fig. 11F) while increased by circTMTC1 overexpression vector (*P* < 0.01; Fig. 11G). Moreover, rescue experiments showed that circTMTC1 can relieve the inhibition of miR-128-3p on MSTN (*P* < 0.05; Fig. 11H).

## Discussion

CircRNAs are rapidly attracting the attention of more and more researchers, and recent studies have established the multiple roles of circRNA in diverse cellular functions and pathways. Muscle-related circRNAs have been found to be widely and differentially expressed in skeletal muscle of animals. The chicken is an important farm animal that serves as a major protein source for humans and an animal model that is used in embryonic muscle development research. Nie *et al*., the first group to study chicken circRNAs, performed RNA-seq on leg muscles of female Xinghua chicken at embryonic day 11 (E11), E16 and 1 day post-hatch (P1) and identified 462 DECs at all three times points, including 236, 285 and 89 circRNAs in E11 vs. E16, E11 vs. P1, and E16 vs. P1 comparison groups, respectively 16. To expand our understanding of the functions of muscle-related circRNAs, we selected standardized broilers and layers for RNA-seq, as these have extremely different speeds of muscle accumulation and a similar genetic background.

In total, 4226 circRNAs were identified from all the sequencing libraries, these circRNAs are widely distributed on all chromosomes; moreover, they showed a wide range of expression level and length. By differential expression analysis, 228 circRNAs were found that differentially expressed between the two chicken lines, and only 43 DECs were identified at multiple time points. In comparison with results of Nie *et al*., we found several additional circRNAs related to muscle biological processes, supporting the validity of our RNA-seq between layer and broiler to identify circRNAs across different developmental stages.

In the present study, we found that circTMTC1 was highly expressed in embryonic chicken breast muscle, and it was expressed significantly higher in layer than in broiler at E10, E13 and E16. Additionally, its expression decreased from E10 to E19 both in layer and broiler. These results suggested circTMTC1 may be a negative regulator for chicken skeletal muscle development. To confirm this hypothesis, we first examined the role of circTMTC1 in SMSCs proliferation. The results showed that (1) the mRNA expression of markers of cell proliferation including CCND1, CCND2, CDK2 and PCNA were significantly higher in circTMTC1-knockdown cells, while opposite effects were observed in circTMTC1-overexpressed cells; (2) EdU incorporation assay showed that EdU-positive cells were increased by circTMTC1 knockdown and decreased by circTMTC1 overexpression; (3) and both cell cycle analysis and CCK-8 assay proved that circTMTC1 reduced cell proliferation rate. These results strongly indicated that circTMTC1 inhibits SMSCs proliferation. We also determined the role of circTMTC1 in skeletal muscle cell differentiation. During myogenic differentiation, knockdown of circTMTC1 resulted in the upregulation of gene expression of MyoD1, MyoG and MyHC, which are crucial regulatory factors in muscle cell differentiation, while levels decreased in cicrTMTC1 overexpression cells. MyoG and Myosin protein levels showed similar changes. Myosin immunofluorescence also confirmed that circTMTC1 inhibited SMSCs differentiation into myotubes.

Competing endogenous RNAs is a vital mechanism and even very few miRNA binding sites can be functionally important. Since the circRNA CDR1as was first reported to affect brain function by binding miR-7 12, increasing numbers of circRNAs have been shown to miRNA sponges to influence biological functions. In recent years, some circRNAs were found to regulate animal skeletal muscle development through sponging different miRNAs. However, to the best of our knowledge, only a few circRNAs, including circSVIL, circFGFR2, circHIPK3 and circRBFOX2s in chicken, have been described. Chen's team found that in bovine primary myoblasts, circLMO7 inhibits cell differentiation and promotes cell proliferation and cell survival by sponging miR-378a-3p 29; circFUT10 inhibits cell proliferation and survival and promotes cell differentiation via binding miR-133a 30; and circFGFR4 promotes cell differentiation through sponging miR-107 31. Zhang et al. showed that circZfp609 can sponge miR-194-5p to sequester its inhibition on BCLAF1 to repress myogenic differentiation in a mouse myoblast cell line 32.

In our research, we found that circTMTC1 was mainly located in the cytoplasm, which suggested circTMTC1 may function in post-transcriptional regulation. We found that circTMTC1 was derived from exon 2-5 of the TMTC1 gene and RNAhybrid showed circTMTC1 harbored one potential biding site for miR-128-3p. Subsequently, we confirmed that miR-128-3p was actually combined with the predicated sites of as validated by dual-luciferase reporter assays, RNA pull down assay and qRT-PCR. With further analysis, we found that chicken circTMTC1 functions as a miR-128-3p sponge at the differentiation stage of SMSCs, and circTMTC1 inhibited the expression of miR-128-3p.

miR-128 is a brain-enriched miRNA that was initially known as the profound effect on tumorigenesis 33, 34, but recent studies showed miR-128 plays an important role in myogenesis. In C2C12 myoblasts cells, miR-128-3p inhibits proliferation but promotes myotube formation by targeting myostatin mRNA 11. In bovine SMSCs, miR-128-3p negatively regulates differentiation and proliferation through repressing Sp1 10. Moreover, reduced miR-128-3p abundance in the chicken induced muscle mass loss 35. In our study, we found that miR-128-3p significantly increased muscle differentiation-related gene expression and positively regulated myotube formation of chicken SMSCs, which exactly opposite to circTMTC1. Furthermore, circTMTC1 blocked the promotion effect of miR-128-3p on SMSCs differentiation.

Myostatin (MSTN) is a famous inhibitor of muscle development, the deletion of MSTN gene can cause the excessive development of muscle in animal body^36^, ^37^. Previous studies have shown that MSTN inhibits chicken SMSCs differentiation^38^, ^39^. In our research, we found that miR-128-3p directly target the 3'UTR of MSTN by qRT-PCR and dual-luciferase reporter assays. Considering that miR-128-3p and MSTN have opposite effect on SMSCs differentiation, we have reason to confirm that MSTN is a target gene of miR-128-3p. Furthermore, we also found circTMTC1 can positively regulate the expression of MSTN, and circTMTC1 can eliminates the inhibition effect of miR-128-3p on MSTN expression level. Together these results suggest that circTMTC1 inhibits chicken SMSCs differentiation by sponging miR-128-3p to relieve its inhibition of MSTN. As for how circTMTC1 inhibits SMSCs proliferation, more research is needed.

In conclusion, we performed genome-wide identification of circRNAs by RNA-seq in broilers and layers, and found that circRNAs are abundant and differentially expressed during chicken embryonic development between the two chicken models. We also identified a novel circRNA, circTMTC1, generated by the TMTC1 gene, which inhibits SMSC differentiation by acting as a sponge of miR-128-3p in chicken.

## Supplementary Material

Supplementary Tables.

Supplementary Figures.

## Figures and Tables

**Table 1:** The top 10 highly and differentially expressed circRNAs

CircRNA ID	Host gene	Junction	Length (nt)	DEC group	Regulation	TPM (mean)
NC_006088.4:85026365|85029755	ABI3BP	Exon31-34	273	E16	Up	16053.56
NC_006101.4:8456630|8458456	LOC771758	Exon2-4	393	E19	Down	12867.64
NC_006088.4:165910013|165913579	NAA16	Exon8-9	203	E13, E16	Up	6145.32
NC_006090.4:104713663|104714974	ITSN2	Exon22-24	413	E16	Up	6058.517
NC_006127.4:28161822|28179362	KDM4C	Exon6-8	292	E13	Down	5706.125
NC_006089.4:1000287|1001591	FAM188B	Exon2-3	329	E10, E19	Down	5488.822
NC_006094.4:4669680|4672014	LRRFIP1	Exon21-24	350	E10	Up	4729.361
NC_006089.4:47799749|47802307	BMPER	Exon4-6	257	E16	Down	3419.76
NC_006088.4:59786491|59792400	TMTC1	Exon2-5	621	E10, E13, E16	Down	3290.922
NC_006090.4:18691190|18692054	MARK1	Exon2-3	258	E19	Up	3156.111
