# Supplementary material for: Circular RNA profiling identified an abundant circular RNA circTMTC1 that inhibits chicken skeletal muscle satellite cell differentiation by sponging miR-128-3p
Source: Int J Biol Sci. 2019 Aug 19;15(10):2265–81. doi: 10.7150/ijbs.36412 (PMC6775300; doi:10.7150/ijbs.36412)
Supplement: Supplementary file 2 — Supplementary Figures. [file ijbsv15p2265s2.pdf]

Supplementary Figures

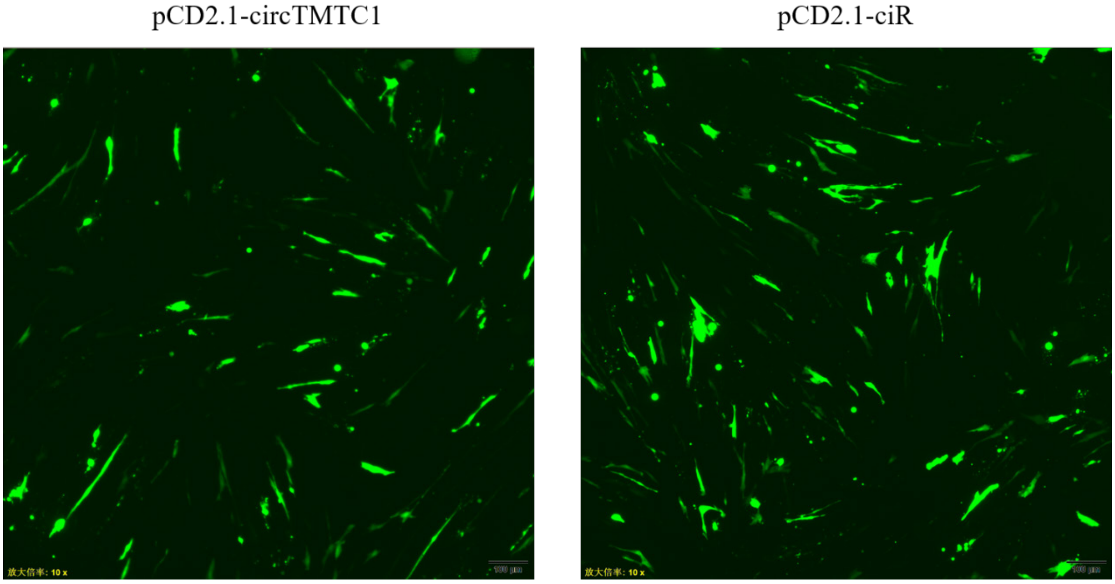

Supplementary Fig. S1. GFP fluorescence of chicken SMSCs after transfected with pCD2.1-circTMTC1 or pCD2.1-ciR vector.

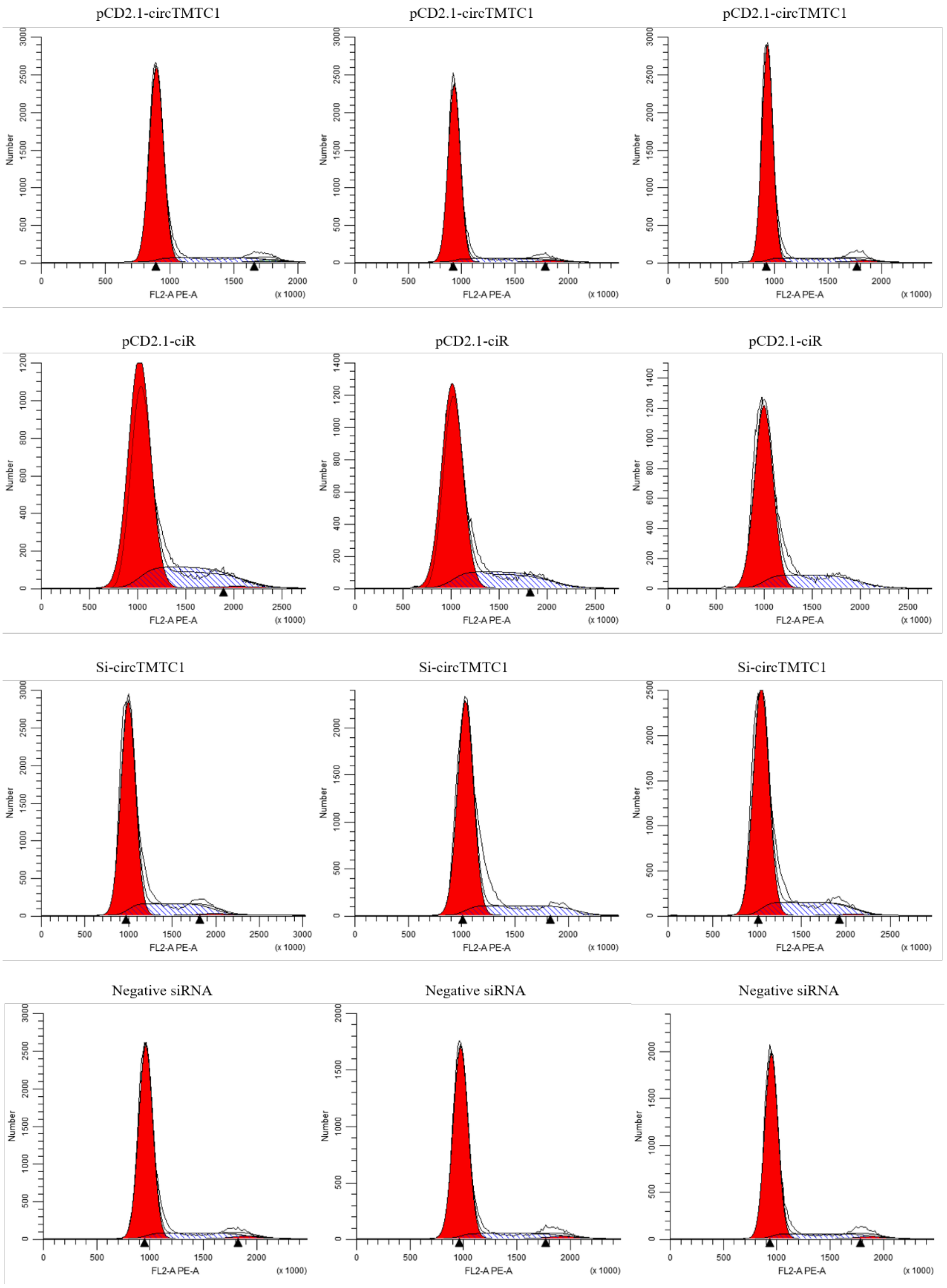

Supplementary Fig. S2. The results of Flow Cytometry of cell cycle assays for chicken SMSCs after transfected with pCD2.1-circTMTC1 vector, pCD2.1-ciR vector, circTMTC1 siRNA or negative siRNA.
